# MSConv-YOLO: An Improved Small Target Detection Algorithm Based on YOLOv8

**DOI:** 10.3390/jimaging11080285

**Published:** 2025-08-21

**Authors:** Linli Yang, Barmak Honarvar Shakibaei Asli

**Affiliations:** 1 College of Mechanical and Electrical Engineering, Nanjing University of Aeronautics and Astronautics, Nanjing 210016, China; linli.yang@cranfield.ac.uk; 2 Faculty of Engineering and Applied Sciences, Cranfield University, Cranfield, Bedford MK43 0AL, UK

**Keywords:** small target detection, MSConv-YOLO, UAV aerial imagery, WIoU

## Abstract

Small object detection in UAV aerial imagery presents significant challenges due to scale variations, sparse feature representation, and complex backgrounds. To address these issues, this paper focuses on practical engineering improvements to the existing YOLOv8s framework, rather than proposing a fundamentally new algorithm. We introduce MultiScaleConv-YOLO (MSConv-YOLO), an enhanced model that integrates well-established techniques to improve detection performance for small targets. Specifically, the proposed approach introduces three key improvements: (1) a MultiScaleConv (MSConv) module that combines depthwise separable and dilated convolutions with varying dilation rates, enhancing multi-scale feature extraction while maintaining efficiency; (2) the replacement of CIoU with WIoU v3 as the bounding box regression loss, which incorporates a dynamic non-monotonic focusing mechanism to improve localization for small targets; and (3) the addition of a high-resolution detection head in the neck–head structure, leveraging FPN and PAN to preserve fine-grained features and ensure full-scale coverage. Experimental results on the VisDrone2019 dataset show that MSConv-YOLO outperforms the baseline YOLOv8s by achieving a 6.9% improvement in mAP@0.5 and a 6.3% gain in recall. Ablation studies further validate the complementary impact of each enhancement. This paper presents practical and effective engineering enhancements to small object detection in UAV scenarios, offering an improved solution without introducing entirely new theoretical constructs. Future work will focus on lightweight deployment and adaptation to more complex environments.

## 1. Introduction

With the rapid advancement of deep learning, object detection algorithms have evolved significantly and found widespread applications across domains such as medical imaging [1], UAV-based target recognition [2], and intelligent traffic monitoring systems [3]. These algorithms are broadly categorised into two types: two-stage and single-stage detection methods [4]. Two-stage approaches typically begin by generating a limited number of high-quality region proposals, which are then refined through classification and bounding box regression [5]. While highly accurate, their computational complexity makes them more suitable for scenarios with low real-time demands, such as medical or satellite imagery [6]. Representative models include R-CNN [7], Fast R-CNN [8], and Faster R-CNN [9].

In contrast, single-stage methods directly predict object categories and locations from image pixels without the need for region proposals. These models employ dense sampling strategies to infer object positions and classes, making them better suited for real-time applications such as autonomous driving [10] and UAV-based detection [11]. Notable representatives include YOLO [12], SSD [13], and DETR [14].

The development of the YOLO family has substantially advanced real-time object detection. In this work, YOLOv8 [15] is adopted as the baseline due to its broad applicability and effective balance between accuracy and inference speed. The architecture divides the input image into grids and simultaneously predicts bounding boxes and class probabilities. However, UAV aerial imagery poses unique challenges, such as the prevalence of small objects, scale variation, occlusion, dense layouts, and complex backgrounds, all of which degrade detection performance and increase the likelihood of missed or incorrect detections [16].

To address these issues, several studies have proposed optimised variants of YOLO. For example, Shi et al. proposed YOLOv5s-2E [17], which utilises K-means++ to recalibrate anchor boxes for the VisDrone dataset and replaces traditional NMS with EIoU-Soft-NMS to better handle occlusions. Additionally, the use of Focal-EIoU accelerates convergence in bounding box regression, and a new detection layer is added alongside DyHead to enhance attention-guided feature integration.

Yang et al. introduced KPEYOLOv5 [18], incorporating three enhancements: optimised anchor generation via K-means++, scSE attention to emphasise small target features within the backbone, and an additional detection head to improve feature extraction for small-scale objects. Similarly, Wang et al. [4] presented an improved UAV detection framework based on YOLOv8, which eliminates unnecessary large target heads, introduces a high-resolution detection branch, and replaces standard convolutions with SPD-Conv for multi-scale feature extraction. GAM attention is integrated into the neck to further boost feature fusion quality.

Li et al. proposed RLRD-YOLO [19], an enhanced YOLOv8-based model featuring a receptive field attention module in the backbone, large-kernel attention to improve spatial pyramid pooling, a reparameterised general feature pyramid neck, and a small-target detection branch—all aimed at improving detection accuracy, particularly for small and dense targets.

Despite these advancements, small object detection remains a significant challenge. To this end, we propose an enhanced model—MSConv-YOLO (MultiScaleConvModule-YOLO)—based on YOLOv8s, with the following key contributions:

- The MSConv module integrates depth-wise separable convolutions, dilated convolutions with varying dilation rates, and a 1×1 convolution for dimensionality reduction. This approach enhances multi-scale feature extraction, improving feature representation without introducing significant computational overhead.
- The original CIoU loss function is replaced with WIoU [20], which is better suited for varying object scales and improves bounding box regression accuracy for small targets in UAV imagery. The contribution lies in integrating this loss function with the MSConv module and the new small object detection layer to enhance the model’s performance for small object detection in UAV scenarios.
- An extra detection head is added to the neck–head structure, operating on high-resolution feature maps to retain fine-grained spatial details, thereby enhancing the detection precision for small objects.
- The combination of these three enhancements (MSConv module, WIoU loss, and additional detection head) provides a more comprehensive solution for small object detection, achieving a balanced optimisation between multi-scale feature extraction, bounding box accuracy, and fine-grained detection precision.

This paper is organised as follows: Section 2 first introduces the network structure of YOLOv8 and then describes the improvements made by different scholars based on this structure for various fields. Section 3 presents the overall structure of MSConv-YOLO and then elaborates on the three improvement points in detail. Section 4 provides a detailed introduction and analysis of the experimental results from multiple aspects, including the dataset, evaluation indicators, comparative tests from different perspectives, and visualisation results. Finally, Section 5 summarises the research achievements of this paper and proposes future improvement directions.

## 2. Related Work

In recent years, the rapid advancement of drone technology has been fueled by their high mobility, fast deployment capabilities, and wide-area surveillance potential. In environments where GPS signals are limited or unavailable—such as densely populated urban settings or remote regions—real-time object detection becomes essential for autonomous navigation and path planning [21]. As a result, object detection algorithms have become a core component of drone navigation systems, where both high-speed inference and robust accuracy are critical [22].

Nevertheless, the elevated operational altitude of drones introduces significant challenges to visual perception tasks. Images captured from such heights are frequently affected by low resolution, background clutter, and unclear target boundaries, all of which hinder detection performance. Despite these issues, recent developments in deep learning-based localisation techniques have led to substantial improvements in detecting small-scale objects within aerial imagery.

Among the various approaches, the YOLO family of algorithms has emerged as a prominent solution, owing to its excellent balance between inference speed and detection accuracy in real-time applications [23]. The following section provides an in-depth examination of the YOLO framework along with recent enhancement strategies tailored for drone-based object detection.

### 2.1. YOLOv8 Network Structure

Developed by Ultralytics, the YOLO series stands as a preeminent single-stage object detection framework, celebrated for its exceptional balance of detection speed and accuracy. Building upon the design merits of YOLOv5 (also developed by Ultralytics) and YOLOv6, YOLOv8 comprehensively enhances the YOLOv5 architecture while retaining its engineering-friendly simplicity. The three primary distinctions between YOLOv8 and YOLOv5 are as follows:

(1) **Transition from Anchor-Based to Anchor-Free:** In anchor-based models like YOLOv5 [24], the network predicts offsets from predefined anchor boxes to adjust positions and classify objects simultaneously. While effective for multi-scale and multi-aspect ratio targets, this approach struggles with small object detection. In contrast, YOLOv8 adopts an anchor-free design [25], which directly predicts object positions through centre keypoint detection and width-height regression. This makes it better suited for detecting small targets.
(2) **Decoupled Head Design:** The coupled head design in YOLOv5 uses a series of convolutional and fully connected layers at the network’s end to simultaneously predict bounding box positions, sizes, and classes across different scales. YOLOv8 replaces this with a decoupled head architecture, which separates classification and regression heads to extract category and location features in parallel [26]. This significantly improves detection accuracy.
(3) **Replacement of C3 Modules with C2f Modules:** YOLOv8 substitutes all C3 modules in YOLOv5 with C2f modules, which feature richer gradient flow. In each bottleneck of the C2f module, the channel dimension of the input tensor is reduced to half of the previous stage, thereby lowering computational costs [27].

**Figure 1:**
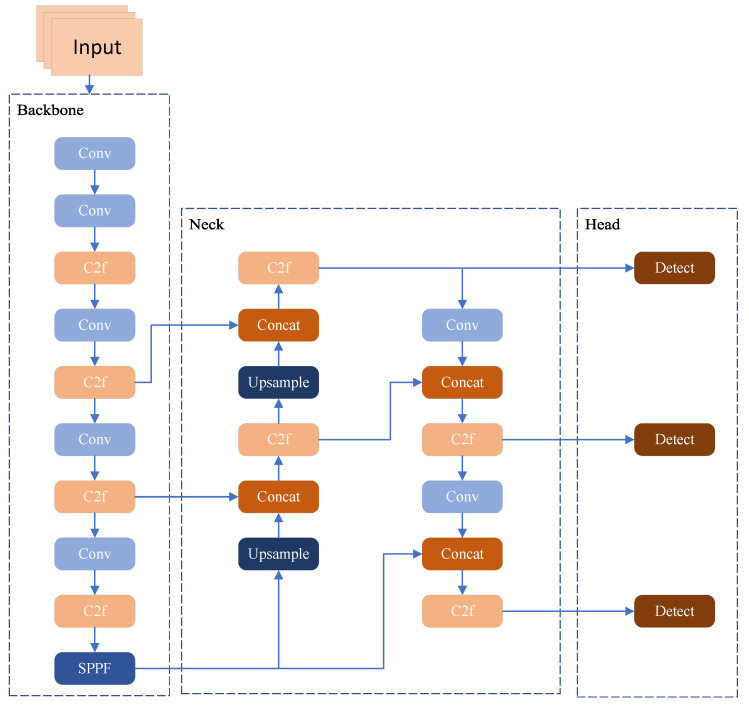
YOLOv8 network structure diagram.

YOLOv8 is available in five sizes: *nano*, *small*, *medium*, *large*, and *extra-large*. To achieve a trade-off between detection performance and inference speed suitable for UAV object detection, this work employs YOLOv8s as the foundational model. As depicted in Figure 1, the YOLOv8 framework is composed of three core modules: *backbone*, *neck*, and *head*. The backbone network is tasked with extracting multi-scale visual features from the input image, converting raw pixels into feature representations with semantic information. After sampling via large convolution kernels, feature hierarchies constructed using the CSP (cross stage partial) structure [28] are employed to build multilayer features; ultimately, the Spatial Pyramid Pooling-Fast (SPPF) [29] module aggregates context information across diverse scales. The neck component serves as a bridge between the backbone and the head, undertaking the fusion of multi-scale features extracted by the backbone. By leveraging structures such as FPN (feature pyramid network) and PAN (path aggregation network) [30], it integrates high-level semantic features with low-level detailed features, thereby enhancing the representation capability of cross-scale contextual information. This feature fusion process effectively alleviates the discrepancy between semantic richness and spatial resolution, laying a solid foundation for subsequent predictive tasks. The head module is dedicated to the final predictive tasks, converting the fused feature maps into specific target information. It typically consists of detection heads and classification heads: the former predicts the spatial coordinates and confidence scores of target bounding boxes, while the latter infers the category probabilities of the detected objects. Through multilayer convolution operations and activation functions, the head module refines the feature representations and outputs the final detection results that meet the task requirements.

### 2.2. Improvement of YOLO Detection Algorithm

The YOLO series of object detection algorithms has achieved notable advancements across diverse industrial domains. Ultralytics, the developer of these frameworks, has continuously iterated and upgraded the algorithms, enabling YOLOv8 to incorporate multi-task capabilities (e.g., detection, segmentation, and classification) and support multiple model sizes. This versatility allows users to select configurations tailored to their specific application requirements. However, in small object detection tasks—characterised by sparse features, significant scale variations, and low pixel occupancy—YOLOv8 still exhibits inherent limitations. In order to address these challenges, researchers have conducted targeted investigations and improvements, which can be categorised into the following three primary directions:

**Backbone Network Improvement:** Wang et al. [31] enhanced YOLOv8’s backbone by integrating the SPD-Conv module into YOLOv8n, improving feature extraction for low-resolution images and small targets, such as defects on railroad tracks. This module replaces strided convolutions and pooling layers with a space-to-depth (SPD) layer and non-strided convolution [32], reducing spatial dimensions while preserving channel information, thereby boosting detection performance for small targets and low-quality inputs. Zhong et al. [33] have improved the SPPF structure in the YOLOv8 backbone by proposing the Sim-SPPF module. This module integrates background and edge information by incorporating AdaptiveMaxPool2d and AdaptiveAvgPool2d, while also introducing the SimAM attention mechanism. It enhances the multi-scale feature capturing ability without significantly increasing computational cost, balancing model accuracy and efficiency.

**Neckwork Network Improvement:** Wang et al. [34] improved the neck structure of YOLOv8 by reconstructing its feature fusion components based on the concept of the Bi-directional Feature Pyramid Network (BiFPN) [35]. They enhanced the feature fusion capability of the neck through bidirectional cross-scale connections and weighted fusion and added new backbone feature paths for dual-input feature maps with the same scale; this improvement strengthens the spatial information of features, thereby boosting the detection accuracy of small targets such as small road cracks. In the NHD-YOLO framework proposed by Chen et al. for PCB surface defect detection, the neck structure enhances YOLOv8’s original PAN-FPN by implementing a shortcut feature pyramid network (SFPN). This design introduces a two-round fusion mechanism for $P_{3},\;P_{4},\;P_{5}$ features using concatenation, depth-wise convolution, and C2f modules, balancing feature distribution to improve the detection of small and complex defects.

**Head Network Improvement:** Wu et al. [36] addressed the limitations of the CIoU loss in PCB defect detection, where small and diverse defect shapes are common. They introduced the PIoU loss, which incorporates an adaptive penalty for target size and a gradient adjustment based on anchor box quality, improving anchor box regression and offering better adaptability to size and shape variations in PCB defects. Lin et al. [37] modified YOLOv8’s head structure by adding a 160×160 small object detection layer to the original three (20×20, 40×40, and 80×80), bringing the total to four. Features from the backbone’s P2–P5 layers, fused via Slim-Neck, feed into this new layer, heightening sensitivity to tiny targets. Though computation sees a slight rise, this tweak bolsters small-object detection and cuts false positives/negatives across scales.

Although numerous studies have made advancements in improving the accuracy and speed of small object detection across various domains, these algorithms still exhibit their respective limitations. Moreover, research on enhancing algorithms for drone-based small object detection in high-altitude operations remains necessary. Therefore, this paper designs a novel structure, MSConv-YOLO, to address this issue.

## 3. Methodology

**Figure 2:**
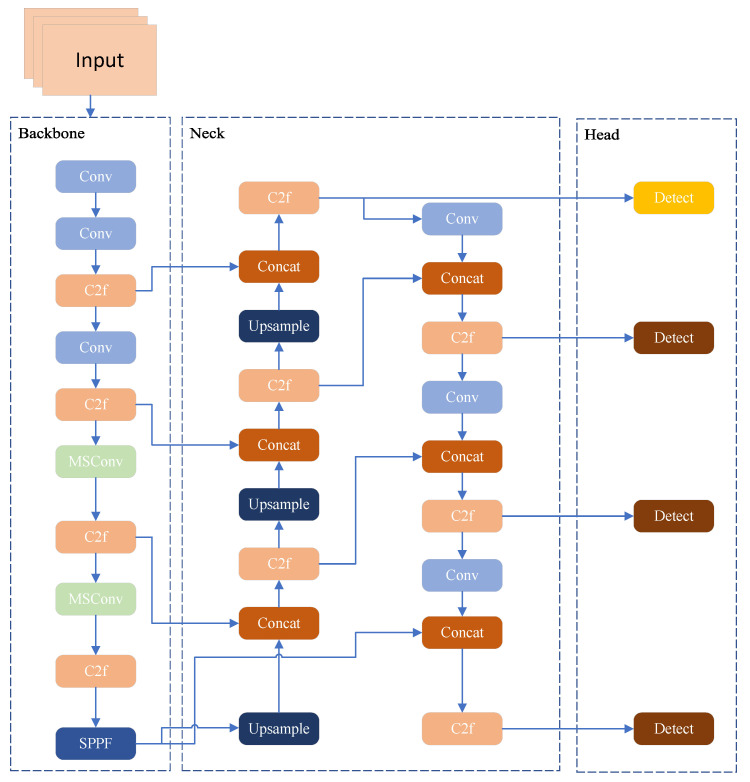
Improved YOLOv8 network structure diagram. The two original standard convolutional modules in the backbone structure have been replaced by MSConv.

To address the challenges of scale heterogeneity, sparse feature representation, and severe background clutter for small target detection in the VisDrone2019 dataset, three pivotal enhancements are introduced to the YOLOv8s architecture. First, standard convolutional blocks in the Backbone are replaced with MSConv (as shown in Figure 2) [38]. This module adopts a parallel-branch design: one branch reduces dimensionality using a ${\mathrm{Conv}}_{1\times 1}$ (1 × 1 convolution), followed by parallel ${\mathrm{DSConv}}_{5\times 5}$ and ${\mathrm{DSConv}}_{7\times 7}$ (depth-wise separable convolutions), to capture fine-grained details and multi-scale structures. The other three branches utilise dilated convolutions (DConv) with dilation rates D=1, D=3, and D=5 to encode contextual information across extended receptive fields [39]. This design enables the simultaneous extraction of fine-scale details, multi-scale structural patterns, and long-range context, thereby enhancing the feature representation for small targets. Second, an extra detection head is added to the neck–head pipeline. Leveraging high-resolution feature maps (e.g., 1/4 of the input resolution), this new head directly captures fine-grained features (such as edges and textures) of small targets, alleviating the “feature dilution” problem in the original architecture and achieving full-scale detection coverage. Third, the CIoU loss is substituted with WIoU. By incorporating a scale-adaptive weight factor, WIoU imposes more significant penalties on bounding box errors for small targets, thus improving the precision of bounding box regression. These improvements synergistically form an optimisation loop of “efficient multi-scale feature extraction → full-scale detection coverage → accurate bounding box regression”, specifically tackling the bottlenecks in small target detection for UAV scenarios.

### 3.1. MultiScaleConvModule—MSConv

**Figure 3:**
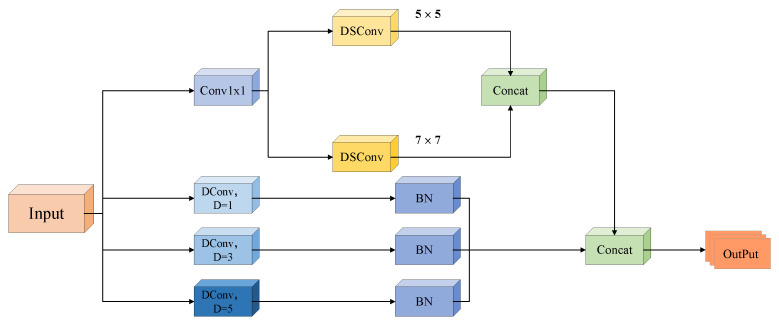
The structure of MSConv.

This module fully exploits the distinct features of depthwise separable convolution, deformable convolution, and dilated convolution and combines the idea of extracting rich features in parallel through multi-scale and multi-type convolutions and then fusing them for output. It extracts small target information in deep networks through four parallel branches. Its structure is divided into two major branches: upper and lower, as shown in Figure 3.

The top branch initiates with a ${\mathrm{Conv}}_{1\times 1}$ to reduce channel dimensionality, striking a balance between computational efficiency and feature preservation. Immediately thereafter, two parallel ${\mathrm{DSConv}}_{5\times 5}$ and ${\mathrm{DSConv}}_{7\times 7}$ are deployed. By decomposing standard convolutions into depthwise spatial filtering (operating independently on each channel) and point−wise channel mixing (via 1×1 convolutions), DSConv expands the receptive field to 5×5 and 7×7 while minimizing parameter overhead.

To quantify this efficiency, we analyse the computational complexity (FLOPs) of DSConv versus standard convolution:

- Standard Convolution: For an input feature map of spatial size H×W, with $C_{\mathrm{in}}$ input channels, $C_{\mathrm{out}}$ output channels, and a $K_{h}\times K_{w}$ convolutional kernel, the computational complexity is:
  (1) $${\mathrm{FLOPs}}_{\mathrm{standard}}=H\times W\times C_{\mathrm{out}}\times K_{h}\times K_{w}\times C_{\mathrm{in}}$$
  This formulation scales quadratically with $C_{\mathrm{in}}$, making standard convolution computationally expensive for deep networks.
- Depthwise Separable Convolution (DSConv): By decoupling spatial filtering (depthwise) and channel mixing (pointwise), DSConv reduces complexity to:
  (2) $${\mathrm{FLOPs}}_{\mathrm{DSConv}}=H\times W\times C_{\mathrm{in}}\times \left(K_{h}\times K_{w}+C_{\mathrm{out}}\right)$$
  Here, the $K_{h}\times K_{w}$ term accounts for depthwise spatial pattern extraction. The $C_{\mathrm{out}}$ term captures point-wise channel fusion. This scales linearly with $C_{\mathrm{in}}$, yielding substantial savings (especially when $C_{\mathrm{in}}\gg 1$, e.g., in deep architectures).

This efficiency is critical for UAV-based small-object detection, where computational resources and real-time inference demands are stringent. The dual-kernel design thus not only captures multi-scale structural details (e.g., edges and textures) of small targets but also aligns with the resource-constrained nature of aerial imaging tasks.

In contrast, the three lower branches utilise dilated convolutions (DConv) with distinct dilation rates D=1, D=3, and D=5, each followed by a batch normalisation (BN) layer. Dilated convolutions introduce spaced sampling between kernel elements, enabling the effective receptive field to expand exponentially (e.g., D=5 yields an 11×11 effective receptive field), without increasing parameter counts or computational complexity. Specifically, D=1 captures fine-grained local adjacencies, preserving small-target details, while D=3 and D=5 encode broader contextual information-critical for distinguishing small targets from cluttered backgrounds in UAV scenes.

- Dilated Convolutions (DConv): Dilated convolutions are designed to increase the receptive field without increasing the number of parameters or the computational cost. By applying gaps between the elements of the kernel, dilated convolutions allow the network to capture long-range dependencies and contextual information without losing resolution or spatial detail. In our model, the varying dilation rates (*D* = 1, *D* = 3, and *D* = 5) allow the network to adapt to different levels of contextual information, which is crucial for identifying small objects at various scales.

While dilated convolutions are effective in enlarging the receptive field, they are known to potentially introduce the “gridding effect” due to their sparse sampling pattern. To mitigate this, MSConv adopts a multi-branch design that combines dilated and non-dilated convolutions. Specifically, the top branch incorporates dense spatial sampling through ${\mathrm{DSConv}}_{5\times 5}$ and ${\mathrm{DSConv}}_{7\times 7}$, which preserve fine-grained local continuity. These undilated paths compensate for the spatial gaps introduced by the lower dilated branches (*D* = 1, 3, 5), ensuring that the module does not lose structural coherence. The fusion of all branches allows the network to simultaneously leverage long-range contextual cues and locally continuous spatial details, effectively balancing contextual expansion and spatial smoothness, and thereby mitigating the gridding effect both theoretically and empirically.

Following multi-scale feature extraction across all four branches, outputs are concatenated to fuse complementary spatial and contextual information. A subsequent channel attention module dynamically reweights channel-wise importance, prioritising discriminative channels (e.g., those rich in edge or texture features of small targets) and suppressing background noise. This adaptive weighting mechanism further amplifies weak small-target signals, addressing their inherent feature sparsity.

Collectively, MSConv addresses three core challenges in UAV small target detection:

- **Scale variation**: Multi-rate dilations and dual-kernel DSConv cover complementary spatial scales, adapting to the diverse sizes of small targets.
- **Feature sparsity**: By fusing fine-grained details (from ${\mathrm{Conv}}_{1\times 1}$ and ${\mathrm{DSConv}}_{5\times 5}$) and contextual cues (from dilated convolutions), the module enriches feature representation for sparse small targets.
- **Efficiency**: Depthwise separable and dilated convolutions ensure the module adds negligible computational cost, preserving YOLOv8’s real-time inference capability. This design philosophy tailors MSConv to the unique demands of small target detection in UAV imagery, laying a robust foundation for subsequent feature propagation in the neck and head modules.

### 3.2. Improved Loss Function

In UAV-based aerial imagery, object detection frequently encounters a predominance of small-scale targets. The choice of an effective loss function is crucial in boosting detection accuracy. YOLOv8 utilises DFL and CIoU for bounding box regression; however, CIoU is not without its drawbacks.

**Figure 4:**
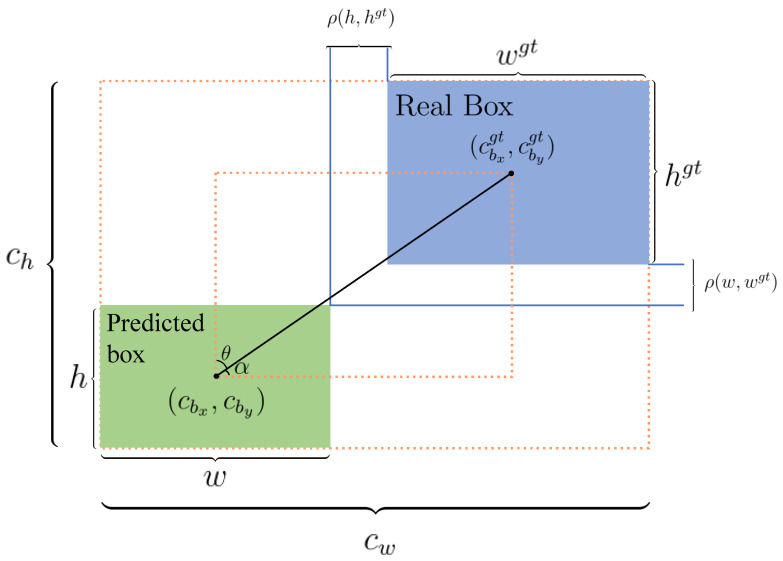
Schematic illustration of the loss function parameters.

To begin with, the aspect ratio component in CIoU is a relative metric. When a predicted bounding box is much larger than the ground truth—despite having the same width-to-height ratio—CIoU fails to adequately penalise the discrepancy in scale, since it is insensitive to absolute size mismatches. This limitation can lead to imprecise localisation. Additionally, CIoU lacks adaptive weighting: all samples, whether difficult (large error) or easy (minor error), are treated identically. As a result, the model cannot focus on challenging cases nor avoid overfitting to simpler ones, leading to a suboptimal optimisation balance. Lastly, CIoU’s reliance on inverse trigonometric functions, such as the arctan term in the aspect ratio penalty, introduces additional computational complexity. The mathematical formulation of CIoU is given in Equation (3):

(3) $$L_{\mathrm{CIoU}}=1-\mathrm{IoU}+\frac{\rho^{2}\left(b,b^{gt}\right)}{{\left(c_{w}\right)}^{2}+{\left(c_{h}\right)}^{2}}+\frac{4}{\pi^{2}}\left(\tan^{-1}\frac{w^{gt}}{h^{gt}}-\tan^{-1}\frac{w}{h}\right),$$

where IoU denotes the intersection-over-union between the predicted bounding box and the ground truth. The variables involved in Equation (3) are illustrated in Figure 4. Specifically, $\rho (b,b^{gt})$ represents the Euclidean distance between the centroids of the predicted and actual boxes. The terms *w*, *h*, and $w^{gt}$, $h^{gt}$ correspond to the width and height of the predicted and ground-truth boxes, respectively, while $c_{w}$ and $c_{h}$ refer to the width and height of the minimum enclosing box that encompasses both boxes.

EIoU addresses some of CIoU’s deficiencies by decoupling the penalties for width and height differences. Unlike CIoU, which merges the aspect ratio into a single penalty term and therefore cannot reflect size differences when ratios match, EIoU introduces explicit penalties for mismatches in both width and height using the Euclidean metric. This refinement enables EIoU to better represent scale discrepancies. The definition of EIoU is provided in Equation (4):

(4) $$L_{\mathrm{EIoU}}=1-\mathrm{IoU}+\frac{\rho^{2}\left(b,b^{gt}\right)}{{\left(c_{w}\right)}^{2}+{\left(c_{h}\right)}^{2}}+\frac{\rho^{2}\left(w,w^{gt}\right)}{{\left(c_{w}\right)}^{2}}+\frac{\rho^{2}\left(h,h^{gt}\right)}{{\left(c_{h}\right)}^{2}},$$

where parameters such as $\rho (w,w^{gt})$ and $\rho (h,h^{gt})$ measure width and height distances, respectively. $\left(c_{b_{x}},c_{b_{y}}\right)$ and $\left(c_{b_{x}}^{gt},c_{b_{y}}^{gt}\right)$ denote the centers of the predicted and ground-truth boxes.

The SIoU loss further innovates by introducing an angular penalty, capturing the rotational alignment between predicted and actual boxes—a factor previously overlooked by CIoU and EIoU. By utilising angular measures (e.g., θ and α in Figure 4), SIoU implements a two-step alignment process: the predicted box first aligns its orientation before fine-tuning its location, thus simplifying optimisation and expediting convergence for objects with significant rotation or orientation variance.

Unlike traditional loss functions such as CIoU and EIoU, which employ static focusing, WIoU offers a dynamic, non-monotonic focusing mechanism, combining classical geometric considerations with adaptive gradient gain allocation. WIoU evaluates anchor box quality during training, adaptively adjusting gradient magnitudes to avoid over-penalising simple samples and emphasising those of intermediate difficulty. According to Tong et al. [20], WIoU has evolved through three iterations:

WIoU v1 adds an attention-based penalty that prioritises spatial deviations only when the predicted box sufficiently diverges from the ground truth, as shown in the following equations:

(5) $$L_{\mathrm{WIoUv}1}=R_{\mathrm{WIoU}}\times L_{\mathrm{IoU}},$$

(6) $$R_{\mathrm{WIoU}}=\exp\left(\frac{{\left(b_{c_{x}}^{gt}-b_{c_{x}}\right)}^{2}+{\left(b_{c_{y}}^{gt}-b_{c_{y}}\right)}^{2}}{c_{w}^{2}+c_{h}^{2}}\right),$$

(7) $$L_{\mathrm{IoU}}=1-\mathrm{IoU}.$$

WIoU v2 further introduces a monotonic focusing coefficient $L^{\mathrm{IoU}}$, normalising it using the mean loss to counteract slow convergence and better downweight easy samples, as detailed in Equation (8):

(8) $$L\mathrm{WIoUv}2={\left(\frac{L^{\mathrm{IoU}}}{L_{\mathrm{IoU}}}\right)}^{\gamma }\times L_{\mathrm{WIoUv}1}.\;\gamma >0.$$

Here, WIoU v3 introduces an outlier indicator β, which, together with the non-monotonic focusing factor *r*, dynamically allocates gradient gains based on anchor quality. A low β (high-quality anchor) results in reduced loss weight, while a high β (poor anchor) yields lower gradients, helping to avoid harmful updates. The v3 formulation is shown in the following equations:

(9) $$L_{\mathrm{WIoUv}3}=r\times L_{\mathrm{WIoUv}1},$$

(10) $$r=\frac{\beta }{\delta \alpha^{\beta -\delta }},$$

(11) $$\beta =\frac{L_{\mathrm{IoU}}^{*}}{\bar{L_{\mathrm{IoU}}}}\in \left[0,+\infty \right).$$

where ∗ denotes the moving average. Based on the above comparative analysis, this work adopts WIoU v3 as the bounding box regression loss. WIoU v3 not only integrates the geometric advantages of EIoU and the spatial adaptability of SIoU but also employs a dynamic, non-monotonic focusing mechanism that encourages the model to prioritise anchors of moderate quality. This dynamic focus is especially vital for UAV-based scenarios, such as those in the VisDrone dataset, where small objects are prevalent and background clutter is substantial. By adaptively tuning loss weights, WIoU v3 significantly enhances the precision and robustness of small object detection, directly addressing the unique challenges presented in aerial imagery.

In addition to performance gains, WIoU v3’s non-monotonic, quality-adaptive gradient allocation influences training convergence by prioritising medium-quality anchors—those with moderate IoU errors—while suppressing gradients from both easy and poor-quality samples. This focus helps accelerate convergence and improve robustness. To avoid instability caused by fluctuations in anchor quality, WIoU v3 scales gradient magnitudes using the outlier indicator β, ensuring that updates reflect actual informativeness. Furthermore, by combining EIoU’s geometric constraints and SIoU’s spatial alignment, the dynamic weighting remains grounded in meaningful spatial metrics, thus maintaining stable and efficient optimisation.

### 3.3. Optimisation of the Prediction Layer

To further enhance the performance and robustness of YOLOv8 in small object detection tasks, researchers have introduced a detection layer as a key optimisation component based on the original structure, effectively alleviating the problem of small object feature loss caused by multiple downsampling operations. Small objects typically exhibit characteristics such as small size, sparse details, and high density, which means that in the original 8×, 16×, and 32× scale feature maps of YOLOv8, they often occupy only a few pixels or may even be completely ignored by convolution operations, thereby severely restricting detection performance. The introduction of the detection layer enables more detailed perception of small objects by connecting to high-resolution feature pathways in the shallower layers of the neck module (e.g., 4× or 8× scales). These shallow feature maps retain rich visual details such as edges, textures, and contours, which are crucial for accurately localising the positions of objects.

Meanwhile, to compensate for the deficiency of shallow features in semantic understanding, the detection layer integrates the FPN (feature pyramid network) and PAN (path aggregation network) mechanisms in the neck structure. It upsamples semantic information from deep abstract semantics, fuses it with shallow details, and constructs a composite representation with “fine-grained structure + high-level semantics”. This fusion not only enhances the recognisability of small objects but also improves the ability to distinguish categories, avoiding misclassification caused by insufficient feature semantics. Furthermore, YOLOv8 adopts an anchor-free detection mechanism, allowing each grid point in the feature map to directly predict the centre point and bounding box. Compared with traditional anchor-based methods, this approach is more suitable for high-density small object scenarios. High-resolution feature maps (e.g., 80×80) provide finer spatial partitioning, making each grid unit cover a smaller actual area, which can more accurately capture the object’s centre point and perform bounding box regression, significantly reducing the risk of prediction conflicts and overlapping misjudgments.

**Figure 5:**
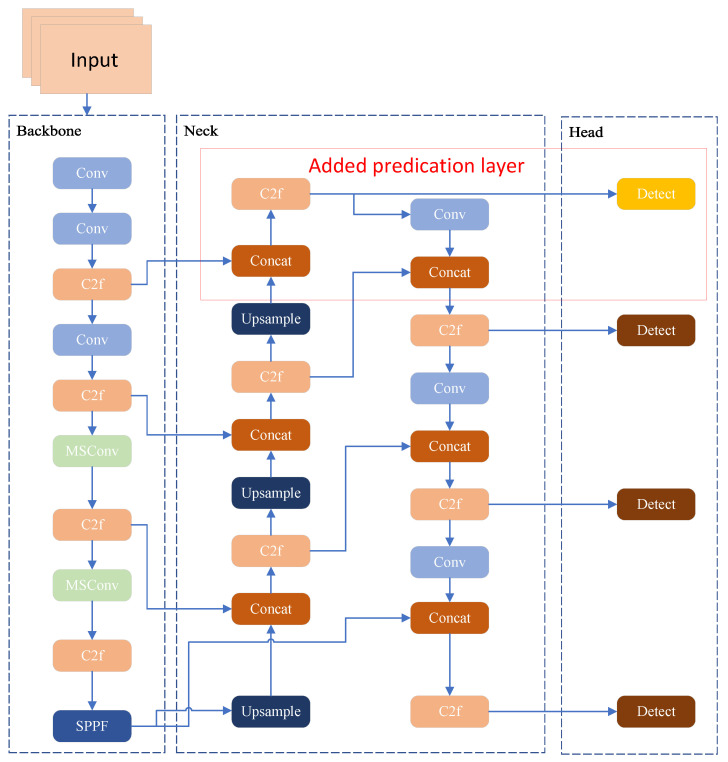
Model after adding prediction feature heads.

Ultimately, with the introduction of the newer detection layer, YOLOv8 constructs an adaptive multi-scale detection architecture: shallow layers detect small objects, leveraging detailed features to enhance localisation accuracy; middle layers detect medium-sized objects, achieving a balance between semantics and structure; deep layers handle large objects, focusing on global semantics and contextual understanding. This multi-level detection system with clear task division not only enhances the adaptability to objects of different sizes but also improves the overall detection accuracy, robustness, and generalisation ability of the model. More importantly, this optimisation strategy maintains the computational efficiency advantages of the original model without significantly increasing the number of parameters or inference latency, making it particularly suitable for application scenarios with high requirements for small object recognition, such as traffic sign recognition, remote sensing image analysis, industrial visual inspection, and intelligent security monitoring. Overall, introducing the detection layer is more than just a simple structural expansion; it also embodies collaborative enhancement at both perceptual and semantic levels, offering an efficient, lightweight, and high-precision solution for small object detection. Figure 5 illustrates the model after prediction feature heads were added to the structure shown previously in Figure 2.

To address the potential risk that high-resolution detection heads may overemphasise spatially local but semantically vague features—particularly when operating on shallow feature maps—the architecture incorporates several design safeguards. First, multi-level feature fusion is achieved through FPN and PAN, where semantically rich deep features are upsampled and integrated with shallow features. This ensures that the detection head receives context-enhanced input, rather than isolated low-level textures. Second, the anchor-free detection mechanism of YOLOv8 allows each spatial location to predict object centers directly, mitigating the influence of noisy background features that typically mislead anchor-based regressors. Third, the use of WIoU v3 as the bounding box regression loss dynamically adjusts gradient emphasis toward medium-quality samples while down-weighting both very easy and unstable ones. This gradient modulation prevents the model from overfitting to superficial patterns and reinforces learning only when spatial detail is accompanied by meaningful semantic context. Together, these mechanisms ensure that the added detection head contributes to fine-grained detection performance without sacrificing semantic reliability or training stability.

## 4. Experimental Preparation and Results

This chapter first outlines the dataset along with the evaluation metrics and hyperparameters employed during training and validation. Subsequently, a comprehensive analysis is carried out based on three experimental comparisons: pre- and post-improvement performance, the proposed model versus other YOLO variants, and ablation studies. These comparisons serve to demonstrate the effectiveness and feasibility of the proposed enhancements. Finally, the model’s performance is further illustrated through visual analyses, including heatmaps and detection result visualisations.

### 4.1. Experiment Introduction

#### Datasets

**Figure 6:**
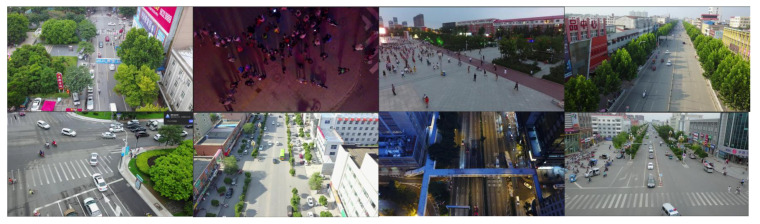
Randomly selected images from the VisDrone-2019 dataset.

To evaluate the effectiveness of the proposed small-object detection architecture, we selected the VisDrone dataset, which includes drone images from fourteen different cities. This dataset captures a wide range of real-world scenarios, covering various weather conditions, times of day, and urban densities, making it a highly representative dataset for testing small object detection in UAV environments (see Figure 6 as some examples).

The VisDrone dataset consists of approximately 10,000 images, and each object is labeled with bounding box coordinates and category labels, including pedestrians, vehicles, bicycles, trucks, and more. The dataset includes diverse scenes from urban, suburban, and commercial areas, with more than 60% of the objects being small in size. The targets are densely packed with frequent overlapping and occlusion, which realistically simulate urban traffic and other complex environments. These characteristics make it an ideal choice for evaluating small object detection algorithms, as it covers the full spectrum of real-world challenges encountered in UAV-based detection tasks.

### 4.2. Experimental Evaluation Metrics

To comprehensively assess the effectiveness of the proposed model, a set of evaluation metrics is adopted. Detection accuracy is quantified using precision, recall, $F_{1}$-score, mAP@50, and mAP@50:95. In parallel, model complexity and computational efficiency are evaluated based on the total number of parameters and floating-point operations per second (FLOPs).

#### 4.2.1. Precision and Recall

Object detection results are primarily categorised into four types: true positive (*TP*), false positive (*FP*), true negative (TN), and false negative (FN). Both precision and recall rely on the quantities of these four types for calculation. Precision represents the proportion of true-positive instances within all instances predicted as positive by the model. It reflects how accurate the model’s positive predictions are. When precision is high, it means that there are relatively few false-positive samples in the set of instances the model deems positive. The formula used to calculate precision is:

(12) $$P=\frac{TP}{TP+FP}.$$

Recall represents the ratio of instances correctly identified as positive by the model to all actual positive instances. It reflects how well the model can capture positive samples. When recall is high, it indicates that the model seldom misses detecting positive instances. The formula used to calculate recall is:

(13) $$R=\frac{TP}{TP+FN}.$$

#### 4.2.2. $F_{1}$-Score

The $F_{1}$-score is the harmonic mean of precision and recall, calculated as:

(14) $$F_{1}=2\times \frac{P\times R}{P+R}.$$

#### 4.2.3. mAP50 and mAP50-95

Mean average precision (mAP) is computed by first determining the precision and recall for each individual class and then taking the average across all classes. mAP50 denotes the average precision calculated when the Intersection over Union (IoU) threshold is set to 0.5. In contrast, mAP50-95 is the average of the average precisions computed over a series of IoU thresholds ranging from 0.5 to 0.95 (typically with an interval of 0.05). The formula for calculating mAP is as follows:

(15) AP=∫P(R)dR,

(16) $$mAP=\frac{1}{N}\sum_{j=1}^{N}AP_{j},$$

where *N* represents the number of classes, and $AP_{j}$ represents the *AP* value for the *j*th class.

#### 4.2.4. Parameters and GFLOPs

The term parameters refers to the total count of learnable weights and biases within a deep learning model. This quantity not only affects the model’s storage footprint but also its learning capacity. Generally, models with more parameters require increased training and inference time.

GFLOPs (giga floating point operations per second) denote the number of floating-point operations, in billions, performed during a single forward pass—for example, when processing an input image. This metric serves as an indicator of the model’s computational load, with higher GFLOPs implying greater hardware requirements for real-time execution.

### 4.3. Experimental Environment and Initial Parameter Settings

All experiments were conducted on a system running Windows 11, with PyCharm 2023.2.1 as the development environment. The implementation was performed using Python 3.9.7 and PyTorch 2.0.0, with CUDA 12.2 support.

The hardware configuration comprised an Intel Core i7-12700K CPU, manufactured by Intel Corporation, headquartered in Santa Clara, CA, USA, and an NVIDIA GeForce RTX 3090 GPU with 16 GB of VRAM, produced by NVIDIA Corporation, also based in Santa Clara, CA, USA. The detailed hyperparameter settings are provided in Table 1.

### 4.4. Experimental Results

#### 4.4.1. Comparison Experiment Before and After Improvement

**Figure 7:**
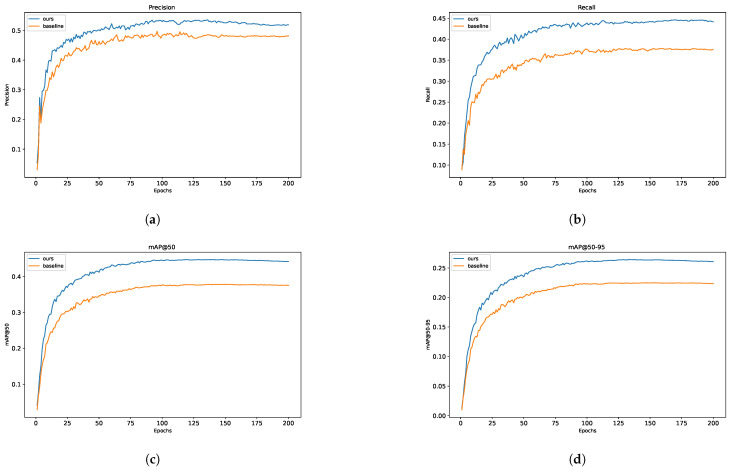
Training curve of YOLOv8s and improved model. (**a**) Precision. (**b**) Recall. (**c**) mAP@50. (**d**) mAP@50-95.

To quantitatively evaluate the enhancement effects, Table 2 demonstrates that our improved model achieves a 6.3% increase in recall and a 6.9% improvement in mAP@0.5 (with IoU≥0.5) compared to YOLOv8s. To intuitively visualise the performance evolution during training, both YOLOv8s and MSConv-YOLOv8 were trained and validated on an identical experimental platform, with the comparative results illustrated in Figure 7. During the initial training stage, MSConv-YOLO exhibited remarkable convergence behaviour, outperforming the baseline YOLOv8s by a substantial margin across all four core evaluation metrics (precision, recall, mAP@0.5, and mAP@0.5:0.95). This finding underscores the model’s enhanced capability in detecting small targets from an unmanned aerial vehicle (UAV) perspective—a scenario where fine-grained feature discrimination and robust localisation are of paramount importance.

**Figure 8:**
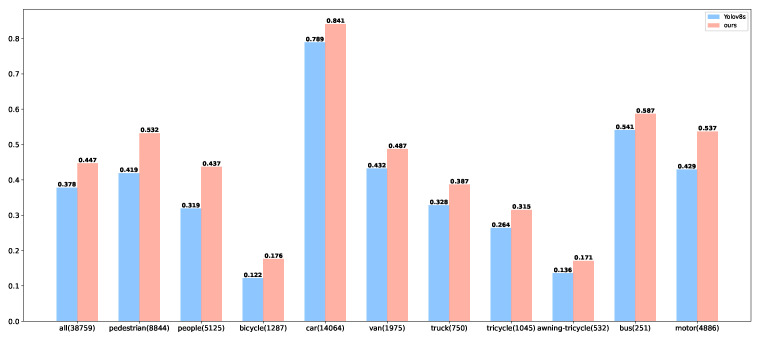
The MAP@50 indicators of the improved and original models.

Table 2 and Figure 7 preliminarily suggest that the MSC-YOLO model outperforms the baseline. To unpack the specific facets of its superiority, we visualise the comparative validation outcomes of MSC-YOLO and the baseline on the VisDrone2019 dataset, as depicted in Figure 8.

The improved model achieves higher detection accuracy across all categories compared to YOLOv8s. Specifically, the global accuracy increases from 37.8% to 44.7%, which demonstrates that the improved module can enhance the overall representational ability of the model. For large sample categories with sufficient samples (i.e., pedestrian, car, and motor), the accuracy of car and pedestrian improved from 78.9% to 84.1% and from 41.9% to 53.2%, respectively. This indicates that the model can stably learn the features of frequent categories, and its bounding box regression is more accurate. Even for small targets in large samples (e.g., pedestrians), the improved model can capture detailed information through more effective feature fusion. Meanwhile, for long-tail categories with scarce samples (i.e., bicycle and awning-tricycle), the improved model achieves breakthrough performance, and the accuracy gain becomes more significant as the amount of data decreases. Specifically, the accuracy of the bicycle increased by 44.3%, from 12.2% to 17.6%; the accuracy of the awning-tricycle improved from 13.6% to 17.1%. These results verify that the proposed model has a strong learning ability for extremely sparse annotations. In the drone scenario, multiple small targets, such as pedestrians and people, exist simultaneously. Moreover, people have the characteristic of being easily obstructed. However, the improved MSC-YOLO model increased the detection accuracy from 31.9% to 43.7%, achieving the highest increase. This clearly indicates that the newly added detection module in the improved module, together with the multi-scale feature convolution module, enhanced the extraction and aggregation of fine-grained features.

In conclusion, building upon the category-level performance analysis, the improved model effectively tackles the core challenges inherent to UAV-based object detection, namely target scale variability and long-tailed category distribution. Through innovative architectural optimisations, it achieves a consistent enhancement in detection accuracy across all object categories, with particularly prominent improvements in small-sample and small-target scenarios. These advancements culminate in robust engineering applicability: in complex real-world contexts such as traffic surveillance and emergency response, the model supports stable deployment without the need for category-specific parameter adjustments, thereby validating its strong generalisation capability and practical utility for UAV-enabled missions.

#### 4.4.2. Comparison with Other Models

To demonstrate the superiority and effectiveness of the improved algorithm proposed in this paper, as well as to explain why YOLOv8s was chosen as the baseline model, we conducted a comparative experiment. In the comparative experiment, we compared the proposed model with several YOLO series algorithms; the results are shown in Table 3. The bold results demonstrate that the proposed model achieves superior accuracy (highest Precision, Recall, $F_{1}$, and mAP) while maintaining a reasonable model size and computational cost that is competitive with other high-performing models.

Early-generation models like YOLOv3-tiny and YOLOv5s, despite their lightweight architectures (e.g., YOLOv5s with 7.2 M parameters), exhibit significantly lower detection accuracy ($F_{1}$: 0.29 and 0.37, respectively) and recall (23.8% and 32.8%) compared to YOLOv8s ($F_{1}$: 0.42 and recall: 37.7%), limiting their applicability in UAV scenarios demanding high-precision detection. While YOLOv6s achieves a competitive $F_{1}$ score of 0.41 and recall of 36.2%, it incurs heavier computational costs with 15.2 M parameters and 36.7G FLOPs (compared to YOLOv8s’ 11.1 M and 28.5 G), reducing its practicality for resource-constrained UAV platforms.

As a more recent model, YOLOv9s demonstrates marginally higher accuracy (38.4% mAP@0.5) and comparable FLOPs (29.7 G) to YOLOv8s, even with fewer parameters (7.2 M). However, YOLOv8s benefits from two years of iterative development, yielding superior ecological maturity (e.g., official UAV-oriented deployment toolchains) and multi-task adaptability. Thus, YOLOv8s strikes an optimal balance between detection performance, computational efficiency, and engineering readiness, making it the ideal baseline for UAV platforms requiring reliable deployment.

In comparison, our proposed model (Ours) outperforms the baseline YOLOv8s and other models in terms of both accuracy and computational efficiency. Our model achieves $F_{1}$ of 0.48, precision (P) of 53.4%, and recall (R) of 44.0%, surpassing YOLOv8s’ $F_{1}$ (0.42), precision (48.1%), and recall (37.%). Additionally, our model achieves a significant improvement in mAP@0.5 (44.%) and mAP@0.5:0.95 (26.4%) while maintaining a reasonable parameter count (15.2 M) and FLOPs (36.7 G).

We also considered more recent YOLO versions and a transformer-based model, RT-DETR, for comparison. Notably, YOLOv10s and YOLOv11s offer improvements in accuracy but come with slightly heavier parameters or computational costs. RT-DETR, a transformer-based model, achieves a higher accuracy of 53.2% for precision (P) and 36.9% for recall (R), but with a lower $F_{1}$ score (0.44) and mAP@0.5 of 35.6%. Despite this, RT-DETR’s computational costs (19.8 M parameters and 38.6 G FLOPs) are considerably higher compared to YOLO-based models.

Table 3 presents a more detailed comparison, highlighting the trade-offs between different models in terms of performance and computational efficiency. The $F_{1}$ score is added to emphasise the balance between precision and recall, providing a more comprehensive view of each model’s performance. Our model (Ours) demonstrates significant improvements in both detection precision and runtime performance, making it highly suitable for real-world UAV-based applications, especially in scenarios requiring both accuracy and real-time speed.

#### 4.4.3. Ablation Experiment

To quantify the contributions of the three proposed modules (MultiScaleConvModule, WIoU, and Detection Layer) to small-target detection in UAV aerial imagery, we conducted ablation experiments with YOLOv8s as the baseline. As presented in Table 4, the first row denotes the baseline YOLOv8s, exhibiting the following metrics: mAP@50=37.8%, mAP@50:95=22.5%, 11.1M parameters, 28.5G FLOPs, and an inference speed of 84 FPS.

When only the *MultiScaleConvModule* was activated (second row), mAP@50 increased to 39.1%, confirming that this module enhances multi-scale feature extraction—essential for handling small targets with varying sizes. The FLOPs remained unchanged, demonstrating the computational efficiency of the design, while the parameter count increased to 15.7M. Interestingly, FPS remained at 84, indicating no major change in the inference speed despite the increase in model parameters.

Introducing *WIoU* (third row) further improved mAP@50 to 39.8% and mAP@50:95 to 23.0%, benefiting bounding-box regression through adaptive gradient allocation. The FPS also remained stable at 84, indicating negligible runtime overhead, as WIoU mainly affects the training phase.

When all three modules were enabled (fourth row), the model achieved a substantial performance boost: mAP@50 reached 44.7% and mAP@50:95 rose to 26.4%. Notably, despite the increase in FLOPs to 36.7G, the FPS decreased to 65, reflecting a slight reduction in inference speed due to the added complexity. This indicates that the proposed architectural enhancements boost detection accuracy, but also introduce additional computational overhead. However, this trade-off is acceptable, as the model provides substantial improvement in precision, which is especially important for real-time UAV applications. Meanwhile, the parameter count slightly decreased to 15.2 M, reflecting structural optimisation.

In summary, the FPS metric complements Params and FLOPs by offering a practical measure of deployment efficiency. The ablation results demonstrate that the proposed modules jointly enhance both the detection precision and runtime performance, making the framework highly suitable for real-world UAV-based applications where both accuracy and speed are essential.

### 4.5. Visual Analysis

#### 4.5.1. Detection Graph Analysis

**Figure 9:**
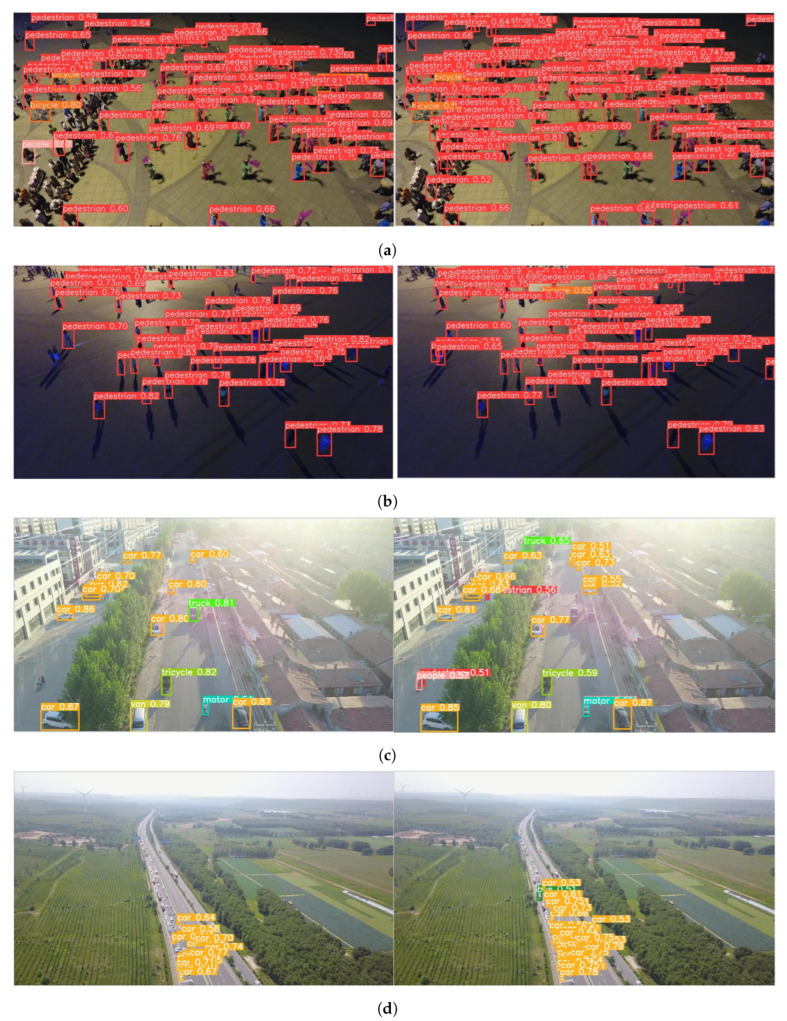
The inference results before and after model improvement on the VisDrone2019 dataset: (**a**) crowded public places, (**b**) dark-light environment, (**c**) bright-light environment, and (**d**) high-altitude road inspection.

Figure 9 illustrates the inference results before and after model improvement on the VisDrone2019 dataset, showcasing the enhanced detection performance across four representative scenarios. In crowded public places (Figure 9a), the improved model reduces missed detections and refines bounding boxes for occluded targets, benefiting from advanced feature fusion that captures fine-grained details. For dark-light environments (Figure 9b), the model detects more small, low-visibility objects with accurate box positioning, indicating a strengthened representation of low-light features. In bright light conditions (Figure 9c), it achieves higher confidence scores and fewer false negatives, demonstrating robustness against illumination-induced distortions. For high-altitude road inspection (Figure 9d), the improved model generates denser and clearer bounding boxes for distant, small targets, reflecting enhanced discriminative feature extraction. These visual results align with quantitative metrics (e.g., accuracy gains for both frequent and long-tail categories), validating the improved module’s efficacy in complex real-world scenarios and complementing numerical performance analysis.

#### 4.5.2. Heat Map Analysis

**Figure 10:**
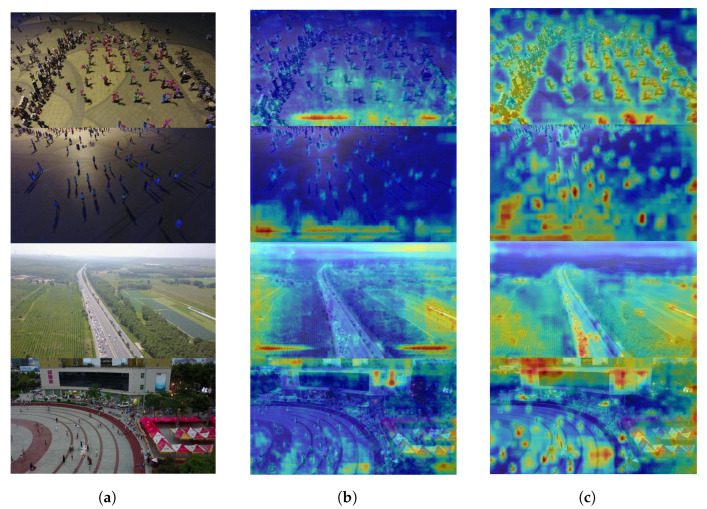
Heat maps: (**a**) original images, (**b**) YOLOv8s, and (**c**) MSC-YOlO.

Figure 10 visualises the heatmaps of original images, YOLOv8s, and MSC-YOLO across diverse scenarios, where the intensity of warm hues (e.g., red, yellow) encodes the magnitude of feature activation. By analysing the spatial distribution of feature responses, distinct improvements in MSC-YOLO’s attention-focusing capability emerge.

In the crowded pedestrian scenario (top row), YOLOv8s displays diffuse activation spanning both background and foreground, while MSC-YOLO concentrates intense responses on pedestrian clusters, even resolving individual targets within dense aggregations. This targeted activation signifies enhanced discriminative ability for small, occluded objects in cluttered environments. For the low-light scene (second row), YOLOv8s generates weak, blurred activation that fails to delineate target boundaries, whereas MSC-YOLO produces sharply defined, target-centred hotspots, demonstrating strengthened robustness to illumination degradation.

In the high-altitude road inspection scenario (third row), MSC-YOLO’s heatmap highlights distant vehicles with precise, concentrated activation—an ability absent in YOLOv8s indistinct response—underscoring improved feature extraction for small-scale targets. For complex urban scenes (bottom row), MSC-YOLO differentiates architectural structures and pedestrian crowds via sharper, semantically aligned activation, contrasting with YOLOv8s’ ambiguous, context-blurring responses.

Collectively, MSC-YOLO heatmaps exhibit more targeted, semantically coherent, and discriminative activation patterns than YOLOv8s. This indicates the improved model optimises feature attention to suppress background noise and accentuate task-relevant features—a critical enhancement underpinning the quantitative gains in detecting small, long-tail, and contextually challenging targets (detailed in Section 3).

## 5. Conclusions

This research focuses on tackling the key issues in small target detection within UAV aerial images—such as scale variation, sparse feature representation, and cluttered backgrounds—by developing MSConv-YOLO, an enhanced version of YOLOv8s. The main improvements are as follows:

First, a new MSConv module is designed, which combines depth-wise separable convolutions with dilated convolutions of different rates. By replacing two standard convolutional blocks in the deeper layers of the backbone, this module strengthens the model’s ability to extract multi-scale features while keeping computational costs in check, effectively capturing both fine details and extended contextual information of small targets. Second, the original CIoU loss is replaced with WIoU v3. This loss function employs a dynamic non-monotonic focusing strategy to prioritise mid-quality anchors adaptively, making it more suitable for the scale changes of small targets in UAV scenarios and improving the accuracy of bounding box regression. Third, a new detection layer is added to the Neck-Head framework. It operates on high-resolution feature maps, which are fused using FPN and PAN mechanisms to retain more fine-grained spatial details, thus reducing the “feature dilution” problem and enabling full-scale detection.

Tests on the VisDrone2019 dataset show that MSConv-YOLO outperforms the baseline YOLOv8s, with a 6.9% rise in mAP@0.5 and a 6.3% improvement in recall. It also achieves consistent performance gains across both common and long-tail categories. Ablation studies further confirm that the three improvements work together to enhance performance, with their combined effect leading to the most notable gains.

The model proves valuable for UAV navigation systems, where real-time and accurate small target detection is essential. However, it has limitations: the increased computational load from the new detection layer and MSConv module may restrict its use on resource-limited devices. Future research will focus on lightweight optimisation, such as model pruning or knowledge distillation, to expand its application to more complex environments. Additionally, exploring transformer-based structures to boost long-range feature connections could further improve detection robustness.

## Figures and Tables

**Table 1:** Hyperparameter settings for training.

Hyper-Parameter	Value
Epoch	200
Batch size	32
Learning rate	0.01
Momentum	0.937
Weight decay	0.0005
Optimiser	SGD

**Table 2:** Comparison of the YOLOv8s and MSC-YOLO metrics.

Model	P/%	R/%	mAP@0.5/%	mAP@0.5:0.95/%	Params/M	FLOPs/G
YOLOv8s	48.1	37.7	37.8	22.5	11.1	28.5
Ours	53.4	44	44.7	26.4	15.2	36.7

**Table 3:** Comparison with other models.

Model	P/%	R/%	$F_{1}$-Score	mAP@0.5/%	Params/M	FLOPs/G
YOLOv3-tiny	37.0	23.8	0.29	22.9	12.1	24.4
YOLOv5s	43.2	32.8	0.37	32.0	7.2	15.8
YOLOv6s	48.5	36.2	0.41	37.1	15.2	36.7
YOLOv8s	48.1	37.7	0.42	37.8	11.1	28.5
YOLOv9s	50.6	36.9	0.43	38.4	7.2	29.7
YOLOv10s	51.0	37.6	0.43	39.0	8.1	24.6
YOLOv11s	49.3	37.6	0.43	38.5	9.2	21.2
RT-DETR	53.2	36.9	0.44	35.6	19.8	38.6
**Proposed Model**	**53.4**	**44.0**	**0.48**	**44.7**	**15.2**	**36.7**

**Table 4:** Ablation experiment.

MultiScaleConvMoudle	Wiou	Detection Layer	mAP@50/%	mAP@50:95/%	Params/M	FLOPs/G	FPS
			37.8	22.5	11.1	28.5	84
✓			39.1	22.8	15.7	28.5	84
✓	✓		39.8	23.0	15.7	28.5	84
✓	✓	✓	44.7	26.4	15.2	36.7	65

## Data Availability

The data presented in this study are available on request from the corresponding author.
